# L-3-n-butylphthalide promotes restoration after an experimental animal model of intracerebral hemorrhage

**DOI:** 10.7150/ijms.60342

**Published:** 2021-04-29

**Authors:** Zhendong Pi, Jianyang Liu, Han Xiao, Zhiping Hu

**Affiliations:** Department of Neurology, Second Xiangya Hospital, Central South University, Changsha, Hunan, China.

**Keywords:** Intracerebral hemorrhage, L-3-n-butylphthalide, Neuroprotection, UBIAD1.

## Abstract

Intracerebral hemorrhage (ICH) is a devastating type of stroke with high morbidity and mortality, and the effective therapies for ICH remain to be explored. L-3-n-butylphthalide (NBP) is widely used in the treatment of ischemic stroke. However, few studies evaluated the therapeutic effects of NBP on ICH. Therefore, the present study aims to evaluate the effects of NBP on ICH and its potential mechanism. The rats were randomly divided into sham-operated group, saline-treated (ICH + saline) group, and NBP-treated (ICH + NBP) group. The ICH model of SD rats induced by IV collagenase was established. The modified Garcia JH score was used to detect the neurological deficit in rats. Western Blot and immunohistochemistry analysis was applied to test the levels of UBIAD1 and caspase-3 expressions in the perihematomal region. The rates of apoptotic cells were detected by TUNEL staining. The results showed that NBP up-regulated the expression of UBIAD1, reduced the apoptotic cells in the perihematomal region, and improved the neurological deficit. Taken together, our study added some new evidence to the application of NBP in ICH treatment.

## 1. Introduction

Intracerebral hemorrhage (ICH), also known as hemorrhagic stroke, is a serious subtype of stroke with high mortality and disability rate 1, 2. The injury mechanism of ICH can be divided into primary injury and secondary injury 2-4. Among them, primary injury refers to brain damage caused by the volume effect of hematoma and physical compression. Strategies to reduce primary injury usually include controlling blood pressure or correcting abnormal blood coagulation to prevent further expansion of hematoma or remove or dissolve blood clots through open and minimally invasive surgery. However, apart from the possible benefits of surgical treatment of ICH, the benefits of surgical treatment have not yet been confirmed in clinical trials in the United States. Secondary injury refers to the physiological reaction of hematoma (mainly edema and inflammation) and the toxic effects of blood components. Strategies to reduce secondary injury include anti-inflammatory reactions such as celecoxib and pioglitazone 5. Reduce the toxic effects of hemoglobin and iron such as iron chelator 6, anti-apoptosis such as citicoline 7. Although the research and trials on the treatment of ICH are increasing, the mortality rate is still high. Hematoma puncture and drug treatment are currently expected by the public, but these methods are still in clinical trials, so there is an urgent need for new effective treatments and drugs.

L-3-n-butylphthalide (NBP) is a drug independently developed in China. A large number of experiments and clinical studies have confirmed that NBP and its derivatives can reduce infarct size 8, protect mitochondrial damage 9, anti-apoptosis 10, antioxidant stress 11, and promote neurogenesis of newborn neurons 12. In addition to being used in the treatment of ischemic stroke, NBP has been proved to be effective in other symptoms or diseases, such as dementia 13-15, Parkinson's disease 16, and Alzheimer's disease 17. Previously, we demonstrate that NBP inhibits the expression of TNF-α and MMP-9, thereby reducing inflammatory reactions, blood-brain barrier damage after ICH 18. However, the possible molecular mechanism of NBP remains further research.

UBIAD1 (UbiA prenyltransferase domain-containing 1), also known as TERE1 (transitional epithelial response gene 1), is located on chromosome 1p36.11-36.33 19, 20. UBIAD1 is are widely found in the brain, lung, heart, liver, kidney, vascular endothelium, colon, bladder, and other tissues. They are indispensable enzymes in the biosynthesis of Vitamin K-2 and CoQ10 21, 22. With the deepening of research, more and more attention has been paid to the role of UBIAD1 in cell metabolism and maintaining internal environment stability 23, 24. UBIAD1 can also regulate the activity of eNOS and play a specific cardiovascular protective role 22, 25. These studies show that UBIAD1 and its catalytic products play an important role in regulating cell proliferation, apoptosis, and oxidative stress, and these mechanisms are closely related to the mechanism of cerebral ischemia-reperfusion and cerebral hemorrhage injury. Previously, we discussed the expression and role of UBIAD1 in an ischemic stroke model, and it has been confirmed that UBIAD1 overexpression has an obvious protective effect on OGD/R-induced cells *in vitro* model 26. However, there is no study to explore the expression and role of UBIAD1 in ICH.

In the present study, the SD rat cerebral hemorrhage model induced by IV collagenase was established. We explored the neuroprotective effect of NBP by measuring the neurological deficit score and cell apoptosis in SD rats after ICH, and further studied the expression and role of UBIAD1 in ICH, which provide a new theoretical basis and direction for the prevention and treatment of ICH.

## 2. Materials and Methods

### 2.1. Experimental Animals and ICH Model

The experiment follows the 3R principle (reduction, replacement, and refinement) put forward internationally for animal experiments, and conforms to the relevant contents of the guiding opinions on being kind to Experimental Animals. All animal experiment procedures are approved by the Animal Experiment Center of Hunan Provincial People's Hospital. A total of 72 healthy male SD rats (weight: 250-300g) were randomly divided into three groups: a sham-operated group (n=24), ICH group (n=24), and ICH + NBP group (n=24). Each group was further randomly divided into four subgroups, named as 6h, 24h, 72h, and 7d subgroups. ICH model was established by injecting IV collagenase into the brain. and then the SD rats of the intervention group were intraperitoneally injected with NBP (25mg/kg, twice a day) 27, and the other two groups were intraperitoneally injected with the same dose of sterilized normal saline. The animals were killed and brain tissues were obtained at each terminal time point.

### 2.2. Neurological Deficit Score

Using the double-blind method, the neurological function of the rats in the three groups was evaluated by the modified Garcia JH method before being killed at each corresponding time point. The neurobehavioral study consisted of the following six tests: Spontaneous Activity, Symmetry in the Movement of Four Limbs, Forepaw Outstretching, Climbing, Body Proprioception, and Response to Vibrissae Touch 28. The minimum neurological score is 3 and the maximum is 18. The lower the score means the more severe the neurological deficit.

### 2.3. Western Blot

The protein was extracted from the tissue around the hematoma with RIPA lysate, and the concentration of the sample protein was detected according to the operation steps of the BCA protein quantitative kit. After the separation gel and concentrated gel are prepared, the sample begins to be electrophoretic and then transferred to a membrane by a transfer apparatus at 300mA for 40 min (caspase3) or 60 min (UBIAD1 and β-actin). The closed membrane was put into the working solution of the primary antibody at 4℃ overnight. Subsequently, the membranes were incubated with the diluted secondary antibodies for 1.5h. Apply ECL chemiluminescence solution (Thermo) evenly on the membranes, incubate for 3 minutes, exposed, and then fixed. The exposed negatives were scanned and protein was visualized by professional grayscale analysis software-quantity one.

### 2.4. TUNEL

The severity of damaged cells around the hematoma was detected by the one step TUNEL apoptosis assay kit (Beyotime Institute of Biotechnology, NanTong, JiangSu, China). Briefly, the paraffin slices were baked in the oven and dewaxed in xylene and alcohol successively. And it placed in sodium borohydride (30min) and Sudan black staining solution (10min), Each sample was added with 50uL endogenous affinity blocking Solution A and B and incubated at room temperature for 20min, and sealed with 5% BSA for 60 minutes, followed by incubation with TUNEL reaction buffer. Being stained by DAPI at 37℃ for 10 min, Finally, the sections were observed by a fluorescence microscope (FluoView FV3000; Olympus Corporation, Tokyo, Japan). In perihematomal region of brain section, we counted the number of DAPI/TUNEL double-positive cells and DAPI-positive cells in 5 regions of each brain section. To evaluate the proportion of injury, we calculated the ratio of DAPI/TUNEL double-positive cells to DAPI-positive cells.

### 2.5. Immunohistochemistry

After dewaxing in xylene and alcohol, the paraffin sections were immersed in citrate buffer to repair the antigen, and then 3% H_2_O_2_ was added to room temperature for 10 minutes. Then, the sections were incubated with primary antibodies (caspase3, UBIAD1) at 4℃ overnight. The secondary antibody was used for incubation for 30min at 37℃. and then the DAB regent was added and incubation at room temperature for 1-5min. The section images were analyzed by Image-Pro-Plus6.0 software.

### 2.6. Data Analysis

All experiments were performed in at least three replicates. Data are expressed as mean ± Standard Error of Mean (SEM). Statistical analyses were performed using SPSS statistical software (SPSS, Inc., Chicago, IL, United States). After testing for normal distribution, the data of two independent variables were analyzed using Mann-Whitney test. For three or more variables, Kruskal-Wallis test was performed followed by post hoc analysis using Tukey's test. Differences with the probability of P < 0.05 were considered significant.

## 3. Results

### 3.1. NBP improves neurological deficits after ICH

**Figure 1:**
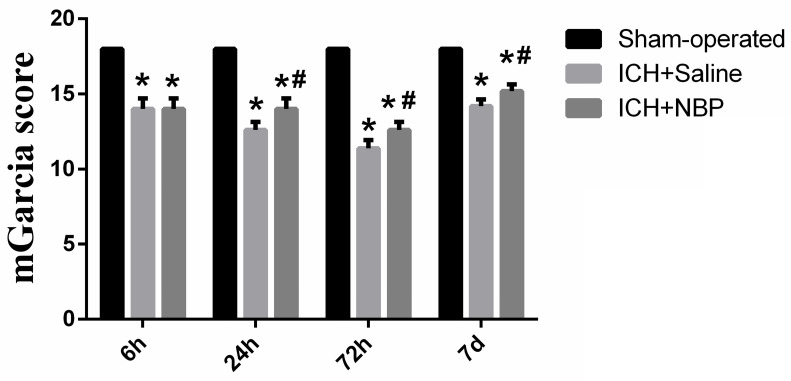
**NBP improves neurological deficits after ICH**. Data were presented as the mean ± SEM (n=5). (^*^P<0.05 vs Sham-operated, ^#^P<0.05 vs ICH + Saline group)

To explore the effect of the NBP on the repair of neurological function, the modified Garcia JH score was performed at 6 h, 24 h, 72h, and 7d after ICH. There was no neurological defect in the sham-operated group. In the ICH group, the neurological deficit score in the 72h subgroup was the lowest, suggesting that the neurological deficit was the most severe at 72h after ICH. Compared to the sham group, the neurological deficit was significantly lower in the ICH group and the ICH+NBP group (p<0.05). The ICH+NBP group demonstrated significantly higher neurobehavioral scores compared to those observed in the ICH group, and there was a significant difference between the ICH+NBP group and the ICH group at 24h, 72h, and 7d (P < 0.05) (**Figure 1**). These results suggested that NBP may effectively repair neurological deficit after ICH.

### 3.2. NBP reduces the apoptosis after ICH

**Figure 2:**
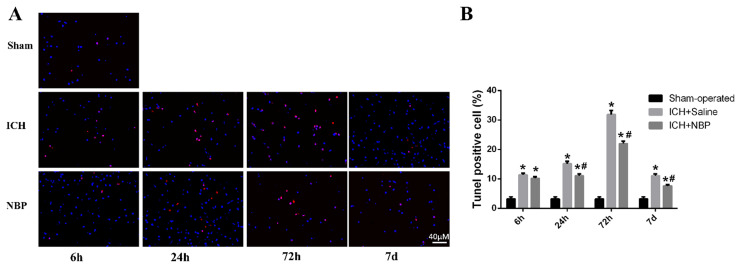
** NBP reduces cell apoptosis in the perihematomal tissue after ICH**. **A**: Cell apoptosis in the perihematomal tissue as detected by TUNEL immunofluorescence staining after ICH. **B:** The ratio of DAPI/TUNEL double-positive cells to DAPI-positive cells. Data were presented as the mean ± SEM (n=3). (^*^P<0.05 vs Sham-operated, ^#^P<0.05 vs ICH + Saline group)

TUNEL staining showed that apoptotic cells were rare in sham group, whereas many apoptotic cells were in ICH group (**Figure 2A**). In the perihematomal region, a large number of the apoptotic cells were observed in the ICH group compared with the sham group. Treatment with NBP shown that it had neuroprotective effects in inhibiting cell apoptosis after ICH. The number of apoptotic cells around the perihematomal tissue in the ICH group was significantly increased than that of the sham group, while the apoptotic cells quantity in the ICH+NBP group was significantly decreased than that of the ICH group at the 24 h, 72h, and 7d after ICH (p<0.05) (**Figure 2B**), suggesting that there was cell apoptosis in the perihematomal tissue following ICH, and NBP reduced the rates of apoptotic cells.

**Figure 3:**
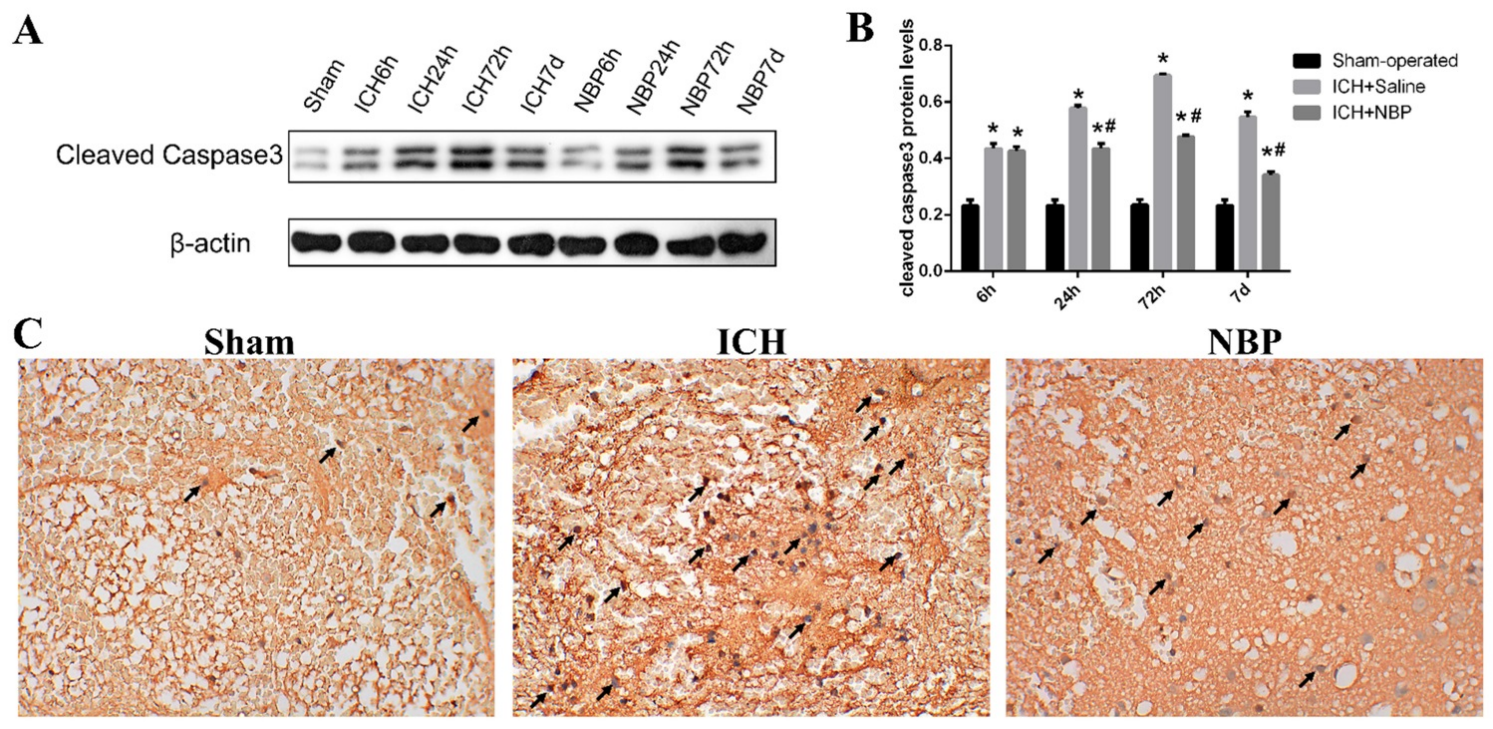
**NBP down-regulated the expression of cleaved caspase-3 in the perihematomal tissue after ICH**. **A**-**B**: The protein expression of cleaved caspase-3 in the perihematomal tissue was detected using Western blotting. **C**: Cleaved caspase-3-positive cells in the cortex of perihematomal region at 72h after ICH. Representative immunohistochemical staining results were shown (magnification × 400). Positive cells were stained brown (black arrow). Data were presented as the mean ± SEM (n=3). (^*^P<0.05 vs Sham-operated, ^#^P<0.05 vs ICH + Saline group)

Cleaved caspase-3 plays an irreplaceable role in the process of cell apoptosis and is the key medium of apoptosis. Apoptosis with increased expression of cleaved caspase-3 can be used as an important marker of ischemic or hemorrhagic injury. To evaluate the effect of NBP on cell apoptosis, we measured the cleaved caspase-3 levels by western blot (**Figure 3A**). In our study, we found that ICH caused a significant increase in the cleaved caspase-3 level compared to those observed in the sham group at the corresponding time point, especially at 72h (p<0.05). While the expression of cleaved caspase-3 in the ICH+NBP group was significantly decreased compared to that in the ICH group (p<0.05) (**Figure 3B**). The expression of cleaved caspase-3 at 72h by immunohistochemistry also revealed the protective effect of NBP on ICH (**Figure 3C**). These results suggest that NBP reduced the expression of cleaved caspase-3 after ICH.

### 3.3. NBP upregulates the expression of UBIAD1 after ICH

**Figure 4:**
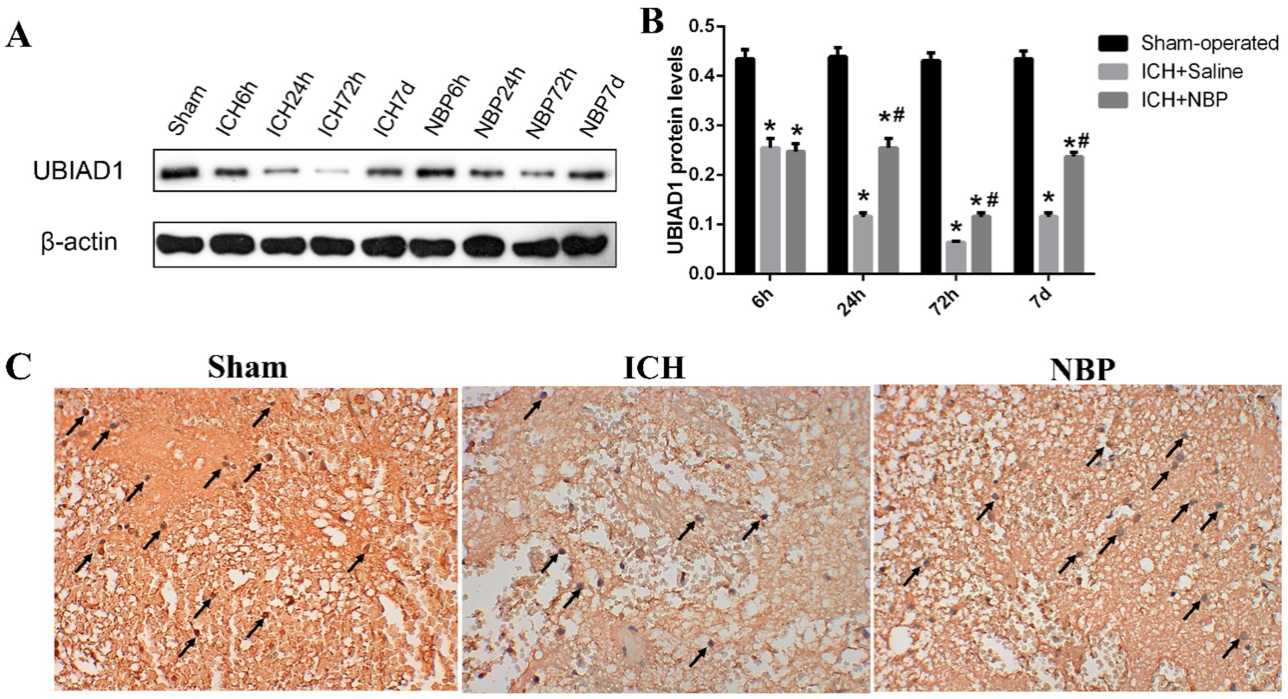
**Expression of UBIAD1 up-regulated by NBP treatment**. **A-B**: The protein expression of UBIAD1 in the perihematomal tissue was detected using Western blotting. **C**: UBIAD1-positive cells in the cortex of perihematomal region at 72h after ICH. Representative immunohistochemical staining results were shown (magnification × 400). Positive cells were stained brown (black arrow). Data were presented as the mean ± SEM (n=3). (^*^P<0.05 vs Sham-operated, ^#^P<0.05 vs ICH + Saline group)

We previously demonstrated that UBIAD1 has neuroprotective effect in ischemic stroke 26. To evaluate the role of UBIAD1 in ICH, we tested the expression of UBIAD1 at different time points after ICH. The results of Western Blot showed that the level of UBIAD1 decreased from 6h, tended to the lowest level at 72h, and then increased gradually in the perihematomal tissue of SD rats. After treatment with NBP, the expression of UBIAD1 was significantly higher than that in the ICH group at 24 h, 72h, and 7d after ICH (**Figures 4A and 4B**). We also performed the expression of UBIAD1 at 72h by immunohistochemistry (**Figure 4C**), which remains the same result as western blot. These results indicated that NBP could up-regulate the expression of UBIAD1.

### 3.4. UBIAD1 has an anti-apoptotic effect

**Figure 5:**
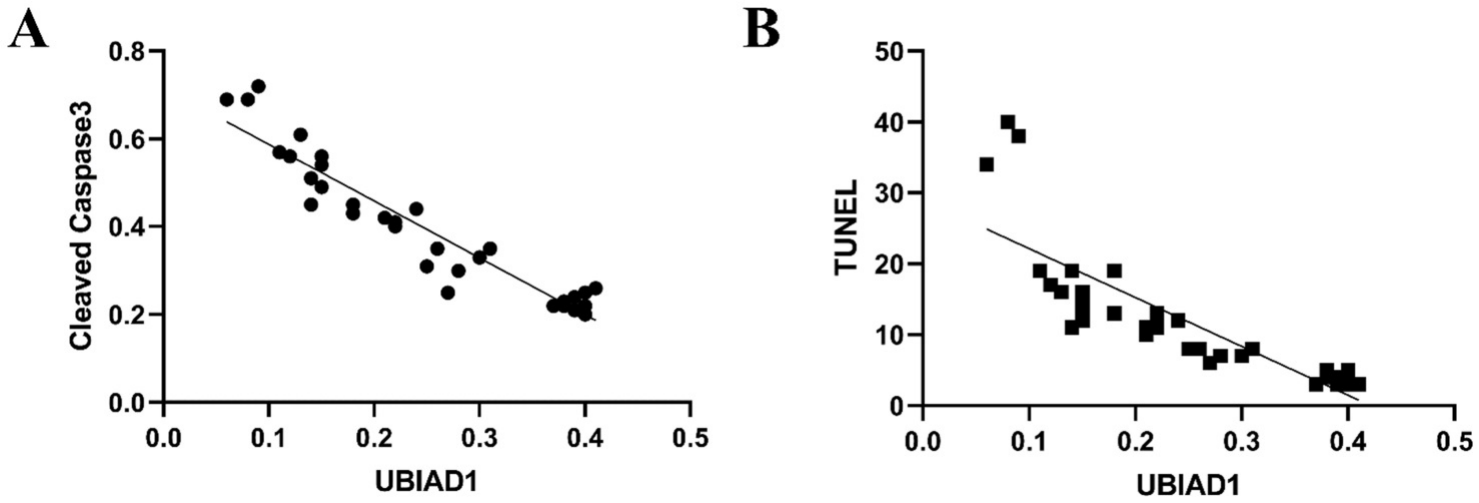
**A**: The negative correlation between the expression of UBIAD1 and Cleaved Caspase-3 after ICH (n=3) (P<0.05). **B**: The negative correlation between the expression of UBIAD1 and the number of TUNEL positive cells after ICH (n=3) (P<0.05)

Some studies have shown that UBIAD1 has both pro-apoptotic and anti-apoptotic effects, which depends on its location, expression, and different pathological stimuli within tissues or cells. Our data showed that cleaved caspase-3 levels increased in perihematomal tissue after ICH, while the expression of UBIAD1 decreased after ICH. After intervention with NBP, the expression of UBIAD1 increased and cleaved caspase-3 levels decreased relatively, suggesting that UBIAD1 may plays an anti-apoptotic role. And there was a negative correlation between the expression of UBIAD1 and the expression of cleaved caspase-3 and the number of TUNEL positive cells after ICH (**Figures 5A and 5B**).

## 4. Discussion

In the present study, we evaluated the neurological function of SD rats after ICH by modified Grace's method, determined the cell apoptosis of perihematomal region by TUNEL staining and cleaved caspase-3 levels. These results showed that the number of positive cells in TUNEL staining and the expression of cleaved caspase-3 were consistent with the changing trend of neurological deficit score in SD rats after ICH, which confirmed that the expression of cleaved caspase 3 could reflect the trend of apoptosis to some extent, and cleaved caspase 3 could be regarded as an effective factor in the process of apoptosis. In the sham operation group, there were a certain number of TUNEL positive cells and a small amount of cleaved caspase-3 expression at each time point, indicating that apoptosis is a normal phenomenon, which is of great significance to maintain the stability of the internal environment and ensure normal growth and development. TUNEL positive cells could be detected at 6 hours after ICH in SD rats, which was significantly higher than that in the sham group (P < 0.05), which indicated that there was an increase of apoptosis at 6 hours after ICH. With the extension of time, the expression of TUNEL positive cells and cleaved caspase-3 reached the peak at 72 hours after ICH, and then decreased, suggesting that 72 hours is the peak time of apoptosis, which overlaps with the peak time of collagenase-induced edema and the lowest neurological deficit score in the present study, suggesting that apoptosis is the cause of neurological dysfunction after ICH. It is one of the main causes of secondary injury after ICH, which is consistent with the conclusions of Dr. Qureshi and other scholars 29. In addition, the expression of TUNEL positive cells and cleaved caspase-3 could still be detected 7 days after ICH, indicating that apoptosis existed in the whole process of ICH. Therefore, reducing cell apoptosis is an effective measure for the treatment of ICH.

The preclinical studies revealed that NBP exerts neuroprotective effects in ischemic stroke both *in vivo* and *in vitro*, partially by promoting angiogenesis 30, increasing BDNF expression 12, promoting dendrite development 31, protecting mitochondrial function 9, inhibiting neuroinflammation 32, and improving brain metabolism 33. The clinical trials also demonstrated that NBP exerts anti-ischemic effects in patients. According to the results of a multicenter phase 2 and phase 3 randomized controlled clinical trials, NBP was approved by the State Food and Drug Administration of China as a drug for the treatment of ischemic stroke in 2002, which has shown good safety and tolerance. A randomized, double-blind, double-dummy trial suggested the 90-day treatment with NBP could improve outcomes at the third month after acute ischemic stroke 34. A meta-analysis revealed that the combination of NBP and standard anti-ischemic stroke drugs is more effective than standard drugs for patients with ischemic stroke 35. However, it is currently uncertain whether or not NBP has a functional role in ICH. Previously, we confirmed that the neuroprotective effect of NBP may be related to inhibiting the expression of TNF-α and MMP-9, and down-regulating oxidative stress and DNA damage after ICH 18. Meanwhile, other studies suggested that NBP can up-regulated the expression of VEGF and angiopoietins-2 (Ang-2) proteins 36, and decreased the expression of AQP4 37. Collectively, the cellular and molecular mechanism of NBP protection in ICH includes promoting neovascularization, attenuating inflammation response, and decreasing brain edema. However, whether other molecular mechanism is involved in the effects of NBP remains elusive.

In the previous study, we demonstrated that the expression of UBIAD1 decreased under the condition of ischemic stroke, and over-expression of UBIAD1 significantly protects against cerebral ischemia/reperfusion-induced neuronal apoptosis 26. Whether UBIAD1 presents similar effects in ICH remains further research. In the present study, the expression of UBIAD1 at different time points after ICH injury was evaluated. Compared with the sham group, the expression of UBIAD1 in peri-hematoma tissue decreased at 6 hours after ICH insult, reached the lowest at 72 hours, and then increased. The changing expression of UBIAD1 suggests that UBIAD1 may be involved in the process of ICH injury, and ICH injury significantly reduces the expression of UBIAD1. The change of UBIAD1 expression was consistent with the degree of neurological deficit symptoms, suggesting that UBIAD1 may be an index to evaluate the severity of neurological impairment after ICH in rats. After intraperitoneal injection of NBP, the expression of UBIAD1 was significantly higher than that in the ICH group at each time point, indicating that the intervention of NBP could up-regulate the expression of UBIAD1.

Moreover, the increased expression of UBIAD1 was accompanied by the decreased expression of cleaved caspase-3, and the decreased number of TUNEL positive cells. We analyzed the correlation between the expression of UBIAD1 and the number of cleaved caspase-3 and TUNEL positive cells. It was confirmed that there was a negative correlation between the expression of UBIAD1 and the expression of cleaved caspase-3, and the number of TUNEL positive cells after ICH, indicating that UBIAD1 showed anti-apoptosis effects after ICH. The neuroprotective mechanism may be the protection of multiple subcellular organelles. Previously, we discovered that UBIAD1 exhibits multi-subcellular organelles colocalization in mouse N2A neuroblastoma cells, including in the mitochondria, endoplasmic reticulum, and Golgi apparatus 26. Up-regulating the expression of UBIAD1 could attenuated cerebral ischemia/reperfusion-induced mitochondrial fragmentation and dysfunction 38, and ameliorated the fragmentation and reduced the level of oxidative stress-related protein expression in both the endoplasmic reticulum and Golgi apparatus.

**Figure 6:**
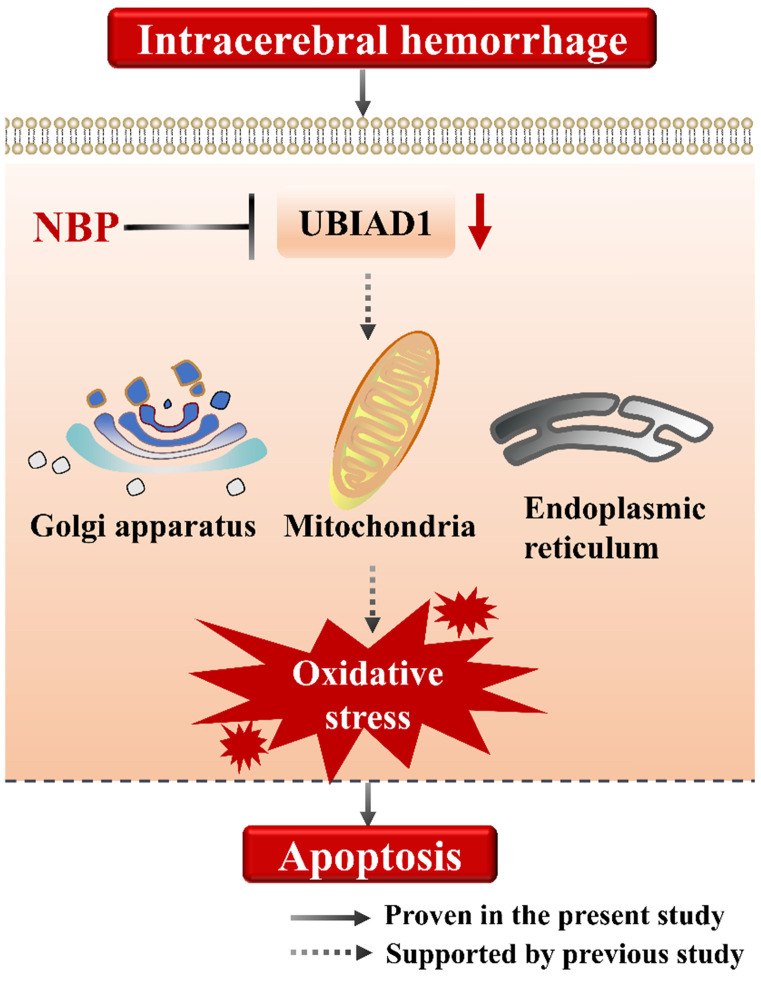
**A schematic representation of the proposed mechanism of NBP therapy in ICH**. In the present study, we suggested that the cell apoptosis and down-regulated UBIAD1 after ICH could be rescued by NBP administration. We previously demonstrated that UBIAD1 could attenuated oxidative stress by protecting the mitochondria, Golgi apparatus, and endoplasmic reticulum. Collectively, NBP therapy is a promising approach for the management of ICH.

Collectively, we suggested that the cell apoptosis in perihematomal tissue after ICH could be rescued by NBP administration, the possible mechanism may be related to the up-regulating the expression of UBIAD1 (**Figure 6**). There were several limitations in this study. First, only modified Garcia JH method applied, which might not be optimal for long-term assessment of neurobehavioral function after ICH. Second, the signaling pathways of UBIAD1 regulating the cell apoptosis in ICH model needs to be further explored. Third, the sample size is small. It would be significant to perform the examination of this treatment in a large cohort for subsequent confirmation.

## 5. Conclusion

Our study in an *in vivo* model provides evidence that NBP exerts neuroprotective effects on ICH.

## Abbreviations

ICH: Intracerebral hemorrhage
NBP: L-3-n-butylphthalide
SEM: Standard Error of Mean
UBIAD1: UbiA prenyltransferase domain-containing 1
